# A Generalized Model for Linear-Periodically-Time-Variant Circulators

**DOI:** 10.1038/s41598-019-45013-5

**Published:** 2019-06-18

**Authors:** Changting Xu, Gianluca Piazza

**Affiliations:** 1 QCT Stargate R&D – Santa Clara, Qualcomm Technologies, Inc., Santa Clara, CA 95051 USA; 2 Department of Electrical and Computer Engineer, Carnegie Mellon University, Pittsburgh, PA 15213 USA

**Keywords:** Electrical and electronic engineering, Applied physics

## Abstract

Magnetic-free non-reciprocity based on linear-periodically-time-variant (LPTV) circuits has received significant research and commercial attention since it could revolutionize wireless communications. LPTV circuits are formed by two main components: linear-time-invariant (LTI) networks and periodically-modulated switches. The modulated switches are the core elements to break the reciprocity of LTI networks. To understand and design LPTV circulators, a universal and intuitive analytical model is required. However, such model does not exist as it is extremely challenging to accurately model and fully understand the LPTV behaviour of energy storage networks. To address this limitation, this work introduces a novel analysis method, which is broadly applicable to any LPTV networks, and validates it experimentally. The novelty of this methodology comes from two main contributions: (1) modelling of the switch as a resistor in parallel with a current-controlled current source; (2) the decomposition of the LPTV network into the linear superposition of two LTI networks. We apply this technique to model the exact behaviour of an LPTV circulator in the frequency domain.

## Introduction

The ever-increasing number of wireless devices drives the need to expand the capacity of wireless communication networks. The network capacity is strongly related to the available radio spectrum, which is very congested. Existing bi-directional communication technologies rely on either time division duplexing (TDD) or frequency division duplexing (FDD). Both are called half-duplexing and do not use the available spectrum to its fullest. The ability to transmit and receive electromagnetic signals simultaneously over the same frequency channel would double the efficiency in the utilization of the radio spectrum, hence increasing the network capacity. This is commonly referred to as in-band full duplexing (IBFD). A key technical challenge for the demonstration of IBFD is the suppression of strong interferers between the transmitter (Tx) and the receiver (Rx) blocks. Significant advancements have been made in suppressing these interferers through analogue and digital circuit cancellation techniques^1–3^. Nonetheless, for the shared-antenna architectures, a key challenge exists in attaining an additional 15–20 dB of isolation right at the RF front-end to relax the design of full-duplexing transceivers. Additionally, this isolation needs to be attained through devices that fit in form factors that are compatible with modern wireless devices. Magnetic-free circulators based on linear-periodic-time-variant (LPTV) circuits^4–31^ are the most promising candidates to implement a solution for this challenge. A circulator is a three-port non-reciprocal device that supports unidirectional power transmission, *i*.*e*., from the Tx to the antenna (Ant) and from the Ant to the Rx, while isolating the Rx from the Tx’s large signal and preventing saturation. Conventionally, non-reciprocity has been achieved via ferrite-based material using the Faraday effect. The resulting devices are bulky, expensive, and incompatible with CMOS technologies^32^. The use of active devices to implement circulators has also been explored^33–35^. However, these implementations suffer from poor linearity and noise performance^36^. On the other hand, LPTV circuits are composed of linear-time-invariant (LTI) networks periodically modulated by a switch (or varactor) matrix. According to the Lorentz theorem, LTI networks are reciprocal by nature, which is mathematically expressed by the use of a symmetric electrical matrix (e.g., admittance matrix, scatter matrix, etc.) to describe the relationship between their ports. The periodic modulation of switches or varactors breaks the time invariance of LTI networks and thus it is possible to generate non-reciprocity. The magnet-free implementation of circulators based on LPTV circuits has shown promise in terms of achieving high linearity and low noise, while overcoming weight, size, and cost limitations of its magnetic counterparts. As shown in Table 1, there are four primary topologies for time-varying non-reciprocal networks: angular momentum biasing (AMB)^4–16,31,37^, phase-shifted N-path filters^20–23^, sequentially-switched delayed lines (SSDL)^17,25,26,28–30^, and distributedly modulated capacitors (DMC)^19,27^. Generally, AMB enables symmetric circulators and requires a modulation frequency that is a small fraction of the filter centre frequency when high quality factor resonators are employed, hence reducing the power consumption of the active modulation process^38^. Moreover, the LTI network modulation could be implemented through switches or varactors. Switches are more attractive because they offer simpler implementation, larger modulation ratio, and easier integration^15^. Therefore, the AMB topology is the focus of this work.

**Table 1:** Summary of non-reciprocal device topologies based on LPTV circuits.

Topologies/Principles	Modulated Components	LTI Network Components
Angular Momentum Biasing (AMB)	Switches	MEMS resonators^4,13,14^ MEMS filters^15,31^
	Varactors	L-C tanks^5–12^ MEMS Resonators^16^
Sequentially-Switched Delay Lines (SSDL)	Switches	Transmission Lines^25,26,28,29^ Acoustic Delay Lines^17,30^
Phase Shifted N-Path Filters	Switches	Transmission Lines^20–23^
Distributedly Modulated Capacitors (DMC)	Varactors	Transmission Lines^19,27^

**Figure 1:**
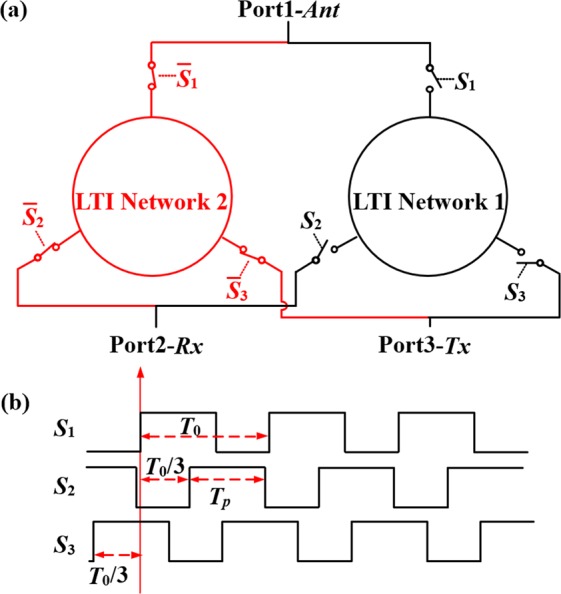
Schematic representation of the generalized non-reciprocal network described herein and the phase difference between modulation signals. (**a**) The proposed non-reciprocal network is formed by two identical LTI networks (1 and 2) with 3-fold rotational symmetry, modulated by two complementary sets of switches. (**b**) There are 120° rotational phase relationships between modulation signals (square wave pulses). *T*_0_ is the modulation period (1/frequency) and *T*_*p*_ is the pulse width. Duty cycle, *α*, is defined by the ratio of *T*_*p*_ to *T*_0_.

Figure 1a shows the proposed general AMB circulator topology^15^. The proposed circulator consists of two identical LTI networks with 3-fold rotational symmetry parametrically modulated by a switch matrix. Figure 1b shows three digital pulse trains with the same period, but a phase difference of 120° with respect to each other. These pulses are the modulation signals used to drive the switches of the black branch of the LTI network. The modulation signals of the red branch complement that of the black one such that the radio frequency (RF) input from the antenna is commutated between the two branches and no power is lost when the switches are in the off state in one branch.

Although the magnet-less non-reciprocal networks have been actively investigated, the analysis of the circuit in Fig. 1 remains challenging due to the complexity of LPTV networks based on modulated switches. Switches are extremely common electronic components; however, it comes as a surprise that, over the last eight decades since sampling mixers came into use in 1930s, the interaction between switches and LTI networks has not been fully modelled. Instead, only a few simple LTI networks modulated by switches have been analysed in either time- or frequency-domain. For example, SSDLs have been qualitatively described in the time-domain by treating transmission lines as non-dispersive elements^17,25,26,30^. Alternatively, sampling mixers have been analysed in the frequency-domain using switch-resistor or switch-capacitor models^38^ where the output and input signals are readily related. More complex models have been developed for N-path filters. E. Klumperink *et al*.^39^ offers a complete overview on the history of N-path filters. In the modelling of N-path filters, assumptions such as loss-less sampling^40,41^ and non-overlapping between switch clock signals^42^ are made to simplify the analysis. These assumptions may offer a good approximation of the circuit behaviour, yet they limit the scope of the methods and are not applicable to a generalized circulator model. The most common techniques to analyse N-path filters are based on polyphase “kernels”^43–45^ and conversion matrices^46^. A kernel is a single-path sub-circuit. This method decomposes the LPTV circuits into multiple polyphase kernels, then each kernel is analysed by invoking LPTV theory, and finally the kernel states are combined to obtain the circuit states. The LPTV theory reveals that^45^, the output spectrum, *V*_*o*_, of an LPTV network, is a summation of an infinite number of frequency-shifted and filtered input spectrum, *V*_*i*_, which is

1 $${V}_{o}(\omega )=\sum _{n=-\infty }^{+\infty }\,{H}_{n}(\omega -n{\omega }_{0})\,{V}_{i}(\omega -n{\omega }_{0})$$

where *H*_*n*_(*ω* − *nω*_0_) is known as “harmonic transfer functions (HTFs)”, *n* is the harmonic index, and *ω*_0_ is the modulation frequency. Finding HTFs is the goal of the analysis of LPTV circuits. The analysis of circulators based on phase-shifted N-path filters has used this kernel method^20,43^. However, such analysis is neither flexible as it cannot handle arbitrary LPTV circuits and arbitrary overlap between clock signals of different kernels, nor intuitive since the results contain an infinite number of translated signals in frequency. Hence, it is quite challenging to analyse a generalized circulator architecture by using polyphase kernels. By contrast, the use of conversion matrices is a more systematic approach, which relies on matrix-form expressions of the HTFs for basic electronic components, such as resistors, capacitors, and inductors. With this method, the analysis of arbitrary LPTV circuits can be performed in a similar way to that of LTI circuits. Nonetheless, it requires to include all explicit component-by-component conversion matrices in the output expression and thus it is not applicable to a generalized LPTV network^46^. A variant of the conversion matrix method was developed to perform the analysis on generalized LPTV networks in ref.^29^. However, it requires to use a large number of square Floquent Scattering Matrices (FSM), which are non-trivial to obtain and make this method more complex. Furthermore, the above methods do not provide a standalone model of a switch by itself without which it remains difficult to fully understand LPTV behaviour.

When varactors are used instead of switches to modulate the LTI networks, then the LPTV circulators can be more readily analysed by coupled-mode theory (CMT) such as in N. A. Estep *et al*.^10^ and R. Fleury *et al*.^47^. This is possible since only first-order intermodulation products have to be considered to obtain an accurate description of the LPTV circulator. However, the proposed models are not applicable to switch based LPTV networks since higher-order mixing must be taken into consideration for accurate description of the circulators.

To address some of the limitations of the aforementioned methods, this work proposes a novel approach to analyse an LPTV network by decomposing it into a finite number of LTI networks and accurately describing its behaviour with semi-analytical equations. This approach is enabled by modelling a periodically modulated switch with a resistor in parallel with a current-controlled current source (CCCS). The resistor represents the on-impedance of the switch, whereas the CCCS current is controlled by the current flowing in the resistor. Such model is independent of the networks the switch connects to and does not impose any restrictions on duty cycles, operating frequencies, and clock overlaps. By using this model of the switch, we are able to separate the description of the switch modulation function from the LTI networks and derive an analytical expression of the proposed generalized circulator as shown in Fig. 1. It is interesting to note that, through this method, the analysis of the LPTV circuit is reduced to the analysis of the comprising LTI circuits in which the switches are effectively on. The circulator model is validated by applying it to the description of a circulator synthesized using off-the-shelf-components. It is worth noting that the same overarching method can be extended to the analysis of any other LPTV circuits with only minor modifications of the component description.

The rest of the paper is organized in the following manner: the switch model in frequency domain is first presented and the interaction between the switch and any arbitrary LTI network is discussed; then such model is used to simplify the description of LPTV networks and derive the semi-analytical S-parameters of the generalized circulator in Fig. 1; finally, the generalized circulator analytical model is validated by comparing its results to the experimental data.

## Standalone Switch Model in Frequency Domain

**Figure 2:**
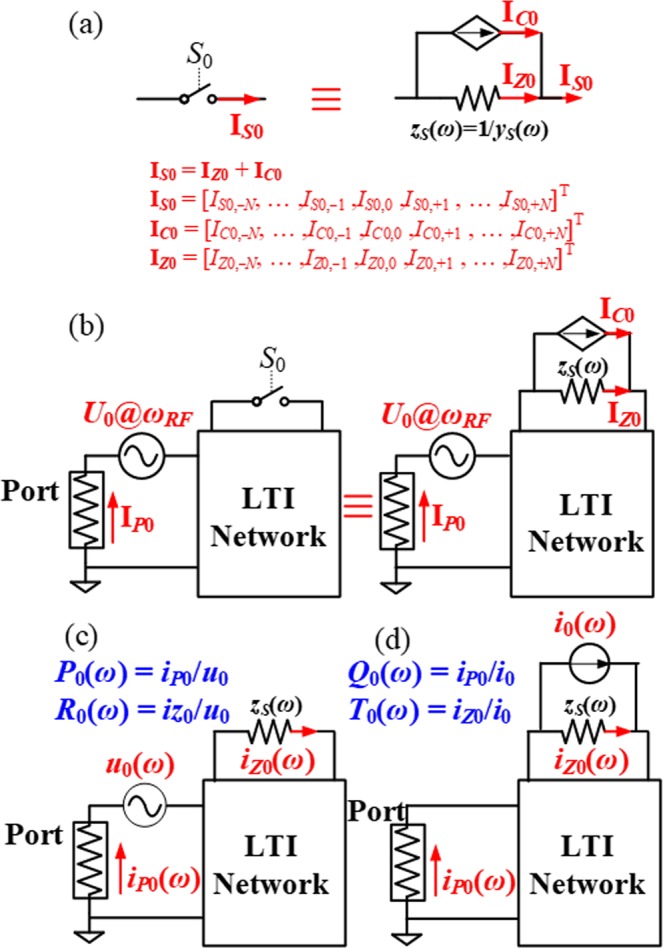
Schematic representation of switch model and its application to a generalized single-switch LPTV circuits. (**a**) Switch model in frequency domain: a resistor in parallel with a current-controlled current source (CCCS). **I**_***S*0**_, **I**_***Z*0**_, and **I**_***C*0**_ are the currents through the switch *S*_0_, equivalent resistor, and CCCS. Each current is a column vector that groups (2*N* + 1) complex current phasors at corresponding frequencies, which are implied by the positions and subscripts of the phasors. For example, *I*_*S*0,*n*_ in **I**_***S*0**_ represents *I*_*S*0,*n*_ exp[*j*(*ω*_*RF*_ + *nω*_0_)]. *z*_*S*_(*ω*) is the spectral on-impedance of the switch, which is inversely related to its on-admittance, *y*_*S*_(*ω*). (**b**) The interaction between a switch and a general LTI network, and its corresponding equivalent circuit. The circuit is driven by a voltage source with internal impedance, *Z*_0_. The source impedance is called “Port”. (**c**,**d**) The two LTI circuits and corresponding transfer functions used to analyse the LPTV circuit in (**b**). The italic lower-case “*i*” means current scalar, while the bold upper-case “**I**” represents current vector, with italic upper-case “*I*” referring to the current elements in the vector.

This work proposes an innovative and illustrative model that describes the dynamic behaviour of a switch periodically toggled on and off using a resistor in parallel with a current-controlled current source (CCCS) (see Fig. 2a and refer to Methods Section for the derivation). The resistor models the frequency dependent (*ω*) on-impedance of the switch, *z*_*S*_(*ω*). The CCCS models the effect of periodic modulation. The current of the CCCS is controlled by the current flowing through the resistor. The quantitative relationship between the CCCS and the current through the resistor is presented in this section and is derived analytically in the Methods Section.

Given a carrier frequency, *ω*_*RF*_, and a modulation frequency, *ω*_0_ (=2*π*/*T*_0_, where *T*_0_ is the period of the modulation signals), the steady-state current through the switch (and the network it connects with) contains intermodulation products at *ω*_*RF*_ ± *nω*_0_, where *n* is an integer. Termed as “harmonic index” in this work, *n* can theoretically span from negative to positive infinity. However, considering that most systems have a finite bandwidth (BW) and that the magnitude of the intermodulation products is proportional to 1/*n*, it is enough to include a finite number of intermodulation products in the analysis. If the maximum harmonic index is *N*, there are (2*N* + 1) frequency components, *i.e*. from (*ω*_*RF*_ − *Nω*_0_) to (*ω*_*RF*_ + *Nω*_0_). Roughly, *N* only needs to be greater than BW/*ω*_0_, which ensures that all effective intermodulation products fall within the band of the system regardless of the relative position of the carrier frequency with respect to the band centre frequency. More broadly, the larger the value of *N*, *i.e*. the number of higher-order intermodulation products included in the analysis, the more accurately the model describes the behaviour of the network. Conversely, when *N* = 0, the number of frequency components is 1 and only the carrier frequency is analysed, which models the network without modulation present (*i*.*e*., the switches are always on).

The LPTV currents generated by periodic switching are represented in the frequency domain by vectors comprised of complex current phasors (frequency information is omitted but implied by the positions and subscripts of phasors), as shown in Fig. 2a. As explained in the Methods Section, the correlation between **I**_*S*0_, **I**_*Z*0_, and **I**_*C*0_ can be expressed as

2 $${{\bf{I}}}_{C0}={\bf{YC}}(\alpha ,\theta ){{\bf{Y}}}^{-1}{{\bf{I}}}_{Z0}$$

3 $${{\bf{I}}}_{S0}={{\bf{I}}}_{Z0}+{{\bf{I}}}_{C0}$$

where *α* is the duty cycle, *θ* is the phase delay of the modulation signal, **Y** is a diagonal spectral admittance matrix of the switch defined in Eq. (33), and **C**(*α*, *θ*) is a (2*N* + 1)-order matrix dictating the mapping relationship in frequency conversion defined in Eq. (34), whose elements are strongly related to the complex Fourier transform coefficients of the switch’s periodic behaviour in time-domain. In the two extreme cases of no modulation, α = 0 or α = 1, **C**(0, *θ*) is a (2*N* + 1)-order negative identity matrix, while **C**(1, *θ*) is a (2*N* + 1)-order zero matrix, which respectively produce:

4 $${{\bf{I}}}_{C0}=-\,{{\bf{I}}}_{Z0},\,{{\bf{I}}}_{S0}=0,\,{\rm{when}}\,\alpha =0$$

5 $${{\bf{I}}}_{C0}=0,{{\bf{I}}}_{S0}={{\bf{I}}}_{Z0},\,{\rm{when}}\,\alpha =1$$

Intuitively, Eq. (4) represents the behaviour of an open switch for zero duty cycle, while Eq. (5) indicates that no modulation is present for 100% duty cycle, *i.e*. the switch is always closed. These two prosperities are independent of *ω*_0_ and *N*.

When the periodically modulated switch interacts with an arbitrary LTI network driven by a voltage source, the switch can be replaced by its equivalent model shown in Fig. 2b. By applying linear superposition theory, the analysis of the circuit in Fig. 2b can be decomposed into the analysis of two LTI circuits as shown in Fig. 2c,d, which consider the contribution of a voltage source (corresponding to the driving source) and a current source (corresponding to the CCCS generated by the switch), respectively. The contribution to the currents at the locations of interest, for example, the port (see Fig. 2b) and the equivalent resistor in this work, can be characterized by four transfer functions, *P*_0_(*ω*), *R*_0_(*ω*), *Q*_0_(*ω*), and *T*_0_(*ω*) as defined in Fig. 2c and d. It is worth noting that the circuit in Fig. 2c is equivalent to the circuit in Fig. 2b without modulation. As proven in the Methods Section, the CCCS pumps currents into the equivalent resistor, which adds to the circuit current without modulation (Fig. 2c) to yield the following steady-state current:

6 $${{\bf{I}}}_{Z0}={{\bf{I}}}_{Z0}^{(0)}+{{\bf{A}}}_{{T}_{0}}{{\bf{I}}}_{C0}$$

where ${{\bf{I}}}_{Z0}^{(0)}$ is the current through the equivalent resistor when the switch is always on, **A**_*T*0_ represents the absorption matrix that depicts the ability of the switch equivalent resistor to absorb current from the CCCS at different intermodulation frequencies, as defined in Eq. (37). Similarly, the modulation modifies the port current, **I**_***P*0**_, in a way that

7 $${{\bf{I}}}_{P0}={{\bf{I}}}_{P0}^{(0)}+{{\bf{A}}}_{{Q}_{0}}{{\bf{I}}}_{C0}$$

where ${{\bf{I}}}_{P0}^{(0)}$ is the current through the port without modulation, **A**_*Q*0_ represents the absorption matrix that depicts the ability of the termination port to absorb current from the CCCS at different intermodulation frequencies, as defined in Eq. (38).

Looking at Eqs (6) and (7), it is interesting to note that the steady-state currents of the resulting LPTV circuits in which the switches are used (Fig. 2b) are composed of two parts: the initial current without modulation and the contribution from the CCCS due to modulation. Therefore, the analysis of the overall current in the LPTV network can be easily conducted by deriving the transfer functions of two LTI circuits as shown in Fig. 2c,d, *i*.*e*. by using *P*_0_(*ω*), *R*_0_(*ω*), *Q*_0_(*ω*), and *T*_0_(*ω*). In summary, the proposed switch model permits treating LPTV circuits (in Fig. 2b) as the linear superposition of two LTI circuits’ states. This point will be illustrated further in the following section.

## Generalized Analysis of the Circulator Circuit

**Figure 3:**
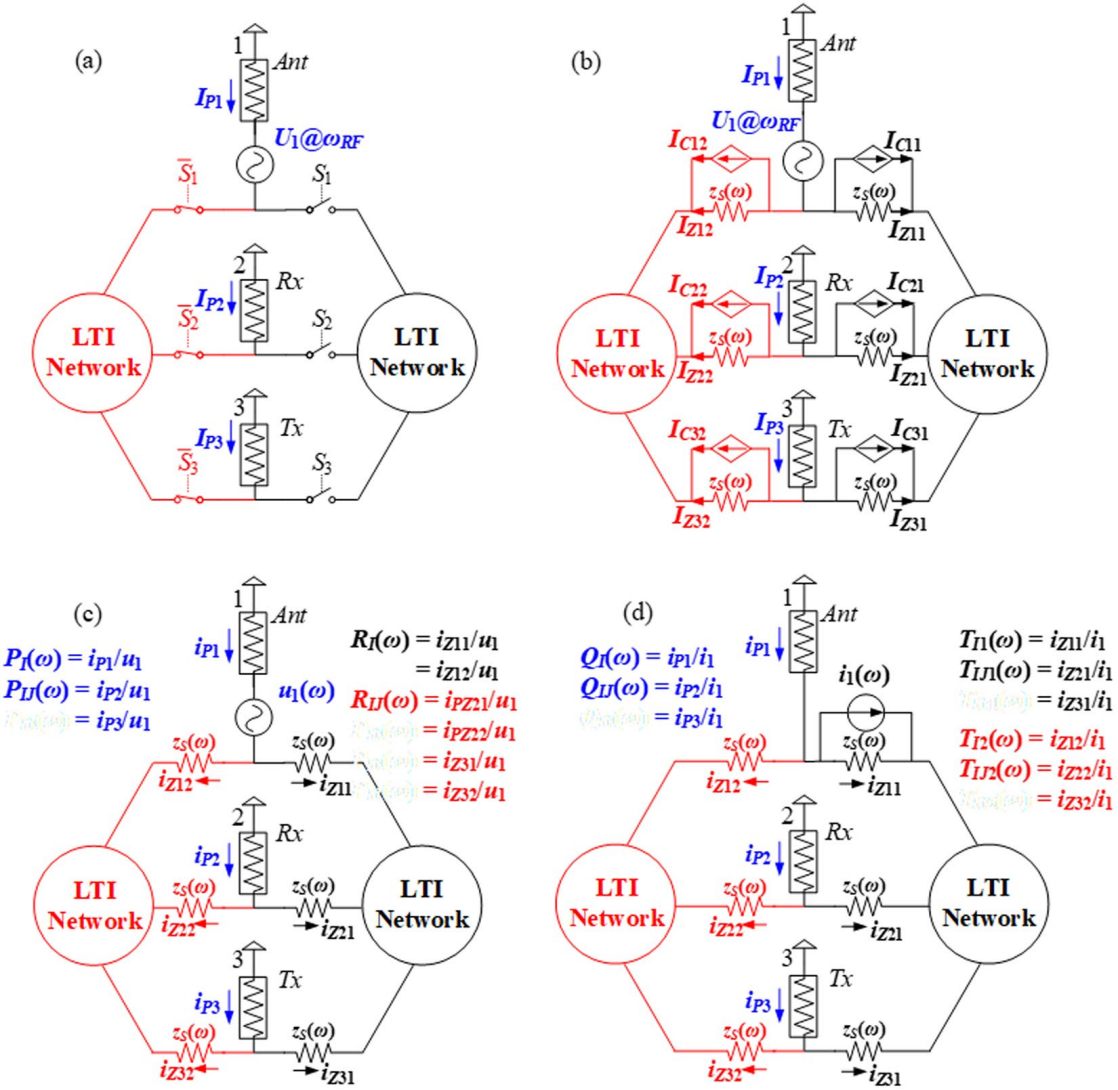
Schematic representation of the proposed methodology for the analysis of the circulator circuit. (**a**) An excitation source is applied at the antenna of the proposed circulator and steady-state port currents, *I*_*P*1-*P*3_, are induced. (**b**) Switches in (**a**) are replaced with *z*_*S*_(*ω*) Ω resistors in parallel with CCCSs according to the equivalent model described in Fig. 2. (**c**,**d**) The two core LTI networks used to analyse the proposed circulator and corresponding transfer functions are defined. *P*_*I*_, *P*_*IJ*_, *R*_*I*_, and *R*_*IJ*_ in (**c**) are used to describe the initial circuit states without modulation, while *Q*_*I*_, *Q*_*IJ*_, *T*_*I*1_, *T*_*IJ*1_, *T*_*I*2_, and *T*_*IJ*2_ help quantify the effect of modulation on the circuit states.

In RF domain, S-parameters are commonly used to describe the network behavior^38^. Our ultimate goal is to derive the closed-form S-parameters of the proposed circulator in Fig. 1a. Due to the 3-fold rotational symmetry of the circulator topology, there are only three independent S-parameters: *S*_11_, *S*_21_, and *S*_31_. Among them, |*S*_21_| is referred to as insertion loss (IL), while |*S*_21_|/|*S*_31_| is called isolation, both being expressed in dB. These two parameters are the most important ones in characterizing the circulator performance. Generally speaking, one wants to minimize IL while maintaining high isolation. To compute the S-parameters, a single-tone voltage source (with carrier frequency, *ω*_*RF*_) excites the circuit at Port 1 (Ant), as shown in Fig. 3a. From the above analysis of the switch, we can replace the switches with resistors and CCCSs (see Fig. 3b). This circuit can be solved by applying linear superposition theory, which corresponds to the analysis of the two core LTI circuits as shown in Fig. 3c,d.

For convenience, we first define *p*- and *r*-transfer functions for the circuit in Fig. 3c:

8 $${P}_{I}(\omega )=\frac{{i}_{P1}}{{u}_{1}},{P}_{IJ}(\omega )=\frac{{i}_{P2}}{{u}_{1}}=\frac{{i}_{P3}}{{u}_{1}}$$

9 $${R}_{I}(\omega )=\frac{{i}_{Z11}}{{u}_{1}}=\frac{{i}_{Z12}}{{u}_{1}},{R}_{IJ}(\omega )=\frac{{i}_{Z21}}{{u}_{1}}=\frac{{i}_{Z22}}{{u}_{1}}=\frac{{i}_{Z31}}{{u}_{1}}=\frac{{i}_{Z32}}{{u}_{1}}$$

as well as *q*- and *t*-transfer functions for the circuit in Fig. 3d:

10 $${Q}_{I}(\omega )=\frac{{i}_{P1}}{{i}_{1}},{Q}_{IJ}(\omega )=\frac{{i}_{P2}}{{i}_{1}}=\frac{{i}_{P3}}{{i}_{1}}$$

11 $${T}_{Ih}(\omega )=\frac{{i}_{Z1h}}{{i}_{1}},{T}_{IJh}(\omega )=\frac{{i}_{Z2h}}{{i}_{1}}=\frac{{i}_{Z3h}}{{i}_{1}},h=1,2.$$

Intuitively, *p*- and *q*-transfer functions describe the fraction of current that each port can absorb from the voltage source and current source, respectively, while *r*- and *t*-transfer functions indicate the respective capability of the voltage source and current source to pump currents into the equivalent resistor. It is interesting to note that, *P*_*I*_(*ω*) and *P*_*IJ*_(*ω*) are related to the S-parameters (${S}_{11}^{(0)},{S}_{21}^{(0)},{S}_{31}^{(0)}$) of the circuit in Fig. 3a without modulation (namely, when switches are always on) in the following way:

12 $$\begin{array}{c}{S}_{11}^{(0)}(\omega )=1-2{P}_{I}(\omega ){Z}_{0},\\ {S}_{21}^{(0)}(\omega )={S}_{31}^{(0)}(\omega )=-\,2{P}_{IJ}(\omega ){Z}_{0},\end{array}$$

where *Z*_0_ is the termination impedance, typically 50 Ω. ${S}_{21}^{(0)}(\omega )={S}_{31}^{(0)}(\omega )$ because of the symmetric circuit topology.

For brevity in the derivation of the circulator model, Table 2 shows the defined naming convention for currents at different locations in the circuit. By using the naming convention, we can re-write equations for each CCCS based on Eq. (1) as:

13 $$\begin{array}{rcl}{{\bf{I}}}_{Ci1} & = & {\bf{YC}}(\alpha ,{\theta }_{i}){{\bf{Y}}}^{-1}{{\bf{I}}}_{Zi1}\\ {{\bf{I}}}_{Ci2} & = & {\bf{YC}}(\overline{\alpha ,{\theta }_{i}}){{\bf{Y}}}^{-1}{{\bf{I}}}_{Zi2}\end{array}$$

where ${\bf{C}}(\overline{\alpha ,{\theta }_{i}})={\rm{C}}(1-\alpha ,2\alpha \pi +{\theta }_{i})$, and *θ*_*i*_ = (*i* − 1)2*π*/3, *i* = 1, 2, 3. Similar to Eqs (6) and (7), the current through equivalent resistors and ports are expressed as the sum of the initial current without modulation and the contribution from CCCSs due to modulation. With the assumption of 3-fold rotational symmetry of the LTI networks, these currents can be expressed as

14 $${{\bf{I}}}_{Zih}={{\bf{I}}}_{Zih}^{(0)}+{{\bf{A}}}_{{T}_{I1}}{{\bf{I}}}_{Cih}+{{\bf{A}}}_{{T}_{I2}}{{\bf{I}}}_{Cil}+{{\bf{A}}}_{{T}_{IJ1}}({{\bf{I}}}_{Cjh}+{{\bf{I}}}_{Ckh})+{{\bf{A}}}_{{T}_{IJ2}}({{\bf{I}}}_{Cjl}+{{\bf{I}}}_{Ckl})$$

15 $${{\bf{I}}}_{Pi}={{\bf{I}}}_{Pi}^{(0)}+{{\bf{A}}}_{{Q}_{I}}({{\bf{I}}}_{Ci1}+{{\bf{I}}}_{Ci2})+{{\bf{A}}}_{{Q}_{IJ}}({{\bf{I}}}_{Cj1}+{{\bf{I}}}_{Cj2}+{{\bf{I}}}_{Ck1}+{{\bf{I}}}_{Ck2})$$

**Table 2:** Naming convention of currents in the proposed circulator topology.

Notation	Meaning
${{\bf{I}}}_{Pi}$	Steady-state current of *i*-th port with modulation, defined as $${{\bf{I}}}_{Pi}={[\begin{array}{ccccccc}{I}_{Pi,-N} & \cdots & {I}_{Pi,-1} & {I}_{Pi,0} & {I}_{Pi,+1} & \cdots & {I}_{Pi,+N}\end{array}]}^{T},$$ where *i* = 1, 2, 3. The current element at the frequency (*ω*_*RF*_ + *nω*_0_) in **I**_*Pi*_ is represented as *I*_*Pi*,*+n*_.
${{\bf{I}}}_{P}$	Steady-state currents of 3 ports with modulation, defined as $${[\begin{array}{ccc}{({{\bf{I}}}_{P1})}^{T} & {({{\bf{I}}}_{P2})}^{T} & {({{\bf{I}}}_{P3})}^{T}\end{array}]}^{T}.$$
${{\bf{I}}}_{Xih}$	Steady-state current of *ih*-th *X* element with modulation, defined as $${{\bf{I}}}_{Xih}={[\begin{array}{ccccccc}{I}_{Xih,-N} & \cdots & {I}_{Xih,-1} & {I}_{Xih,0} & {I}_{Xih,+1} & \cdots & {I}_{Xih,+N}\end{array}]}^{T},$$ where *X* represents either *Z* (switch equivalent resistor), or *C* (CCCS), *i* = 1, 2, 3, and *h* = 1, 2. The current element at the frequency (*ω*_*RF*_ + *nω*_0_) in **I**_*Xih*_ is represented as *I*_*Xih*,+*n*_.
${{\bf{I}}}_{X}$	Steady-state currents of 6 *X* elements with modulation, defined as $${[\begin{array}{cccccc}{({{\bf{I}}}_{X11})}^{T} & {({{\bf{I}}}_{X12})}^{T} & {({{\bf{I}}}_{X21})}^{T} & {({{\bf{I}}}_{X22})}^{T} & {({{\bf{I}}}_{X31})}^{T} & {({{\bf{I}}}_{X32})}^{T}\end{array}]}^{T}.$$
${{\bf{I}}}_{Pi}^{(0)}$	Steady-state current of *i*-th port without modulation, defined as $${{\bf{I}}}_{Pi}^{(0)}={[\begin{array}{ccccccc}{I}_{Pi,-N}^{(0)} & \cdots & {I}_{Pi,-1}^{(0)} & {I}_{Pi,0}^{(0)} & {I}_{Pi,+1}^{(0)} & \cdots & {I}_{Pi,+N}^{(0)}\end{array}]}^{T},$$ where *i* = 1, 2, 3. All components are zero except its element at the carrier frequency. The current element at the frequency (*ω*_*RF*_ + *nω*_0_) in ${{\bf{I}}}_{Pi}^{(0)}$ is represented as ${I}_{Pi,+n}^{(0)}$.
${{\bf{I}}}_{P}^{(0)}$	Steady-state currents of 3 ports without modulation, defined as $${[\begin{array}{ccc}{({{\bf{I}}}_{P1}^{(0)})}^{T} & {({{\bf{I}}}_{P2}^{(0)})}^{T} & {({{\bf{I}}}_{P3}^{(0)})}^{T}\end{array}]}^{T}.$$
${{\bf{I}}}_{Zih}^{(0)}$	Steady-state current of *ih*-th switch’s equivalent resistor without modulation, defined as $${{\bf{I}}}_{Zih}^{(0)}={[\begin{array}{ccccccc}{I}_{Zih,-N}^{(0)} & \cdots & {I}_{Zih,-1}^{(0)} & {I}_{Zih,0}^{(0)} & {I}_{Zih,+1}^{(0)} & \cdots & {I}_{Zih,+N}^{(0)}\end{array}]}^{T},$$ where *i* = 1, 2, 3 and *h* = 1, 2. All components are zero except its element at the carrier frequency. The current element at the frequency (*ω*_*RF*_ + *nω*_0_) in ${{\bf{I}}}_{Zih}^{(0)}$ is represented as ${I}_{Zih,+n}^{(0)}$.
${{\bf{I}}}_{Z}^{(0)}$	Steady-state currents of 6 switches’ equivalent resistors without modulation, defined as $${[\begin{array}{cccccc}{({{\bf{I}}}_{Z11}^{(0)})}^{T} & {({{\bf{I}}}_{Z12}^{(0)})}^{T} & {({{\bf{I}}}_{Z21}^{(0)})}^{T} & {({{\bf{I}}}_{Z22}^{(0)})}^{T} & {({{\bf{I}}}_{Z31}^{(0)})}^{T} & {({{\bf{I}}}_{Z32}^{(0)})}^{T}\end{array}]}^{T}.$$

where *i*, *j*, and *k* are the permutation of 1, 2, and 3, *h* and *l* are the permutation of 1 and 2, and **A**_*X*_ represents the absorption matrix that depicts the ability of ports or switch equivalent resistors to absorb current from CCCS, and it is written as

16 $$\begin{array}{rcl}{{\bf{A}}}_{X} & = & {{\bf{A}}}_{X}({\omega }_{RF},{\omega }_{0})\\  & = & diag[X({\omega }_{RF}-N{\omega }_{0}),\,\ldots \,,\,X({\omega }_{RF}-{\omega }_{0}),\,X({\omega }_{RF}),\,X({\omega }_{RF}+{\omega }_{0}),\,\ldots ,\,X({\omega }_{RF}+N{\omega }_{0})]\end{array}$$

where *X*(*ω*) represents *t-* or *q-*transfer functions. By rewriting Eqs (13–15) in a compact form, we obtain:

17 $${{\bf{I}}}_{C}=\tilde{{\bf{Y}}}\tilde{{\bf{C}}}{\tilde{{\bf{Y}}}}^{-1}{{\bf{I}}}_{Z}$$

18 $${{\bf{I}}}_{Z}={{\bf{I}}}_{Z}^{(0)}+{\tilde{{\bf{A}}}}_{T}{{\bf{I}}}_{C}$$

19 $${{\bf{I}}}_{P}={{\bf{I}}}_{P}^{(0)}+{\tilde{{\bf{A}}}}_{Q}{{\bf{I}}}_{C}$$

where

20 $$\tilde{{\bf{C}}}=diag\,[{\bf{C}}\overline{(\alpha ,{\theta }_{1})}\,{\bf{C}}(\alpha ,{\theta }_{2})\,{\bf{C}}\overline{(\alpha ,{\theta }_{2})}\,{\bf{C}}(\alpha ,{\theta }_{3})\,{\bf{C}}\overline{(\alpha ,{\theta }_{3})}],$$

21 $$\tilde{{\bf{Y}}}=diag[\begin{array}{cccccc}{\bf{Y}} & {\bf{Y}} & {\bf{Y}} & {\bf{Y}} & {\bf{Y}} & {\bf{Y}}\end{array}],$$

2223

The same sub-matrices in matrix ${\tilde{{\bf{A}}}}_{T}$ and ${\tilde{{\bf{A}}}}_{Q}$ have the same colours to help the reader recognize how these two matrices are arranged. Combining Eqs (17) and (18), **I**_*Z*_ can be calculated as

24 $${{\bf{I}}}_{Z}={[{\tilde{{\bf{I}}}}_{d}-{\tilde{{\bf{A}}}}_{T}\tilde{{\bf{Y}}}\tilde{{\bf{C}}}{\tilde{{\bf{Y}}}}^{-1}]}^{-1}{{\bf{I}}}_{Z}^{(0)}$$

where ${\tilde{{\bf{I}}}}_{d}$ is a 6(2*N* + 1)-order identity matrix. Substituting Eqs (24) and (17) into Eq. (19) produces

25 $${{\bf{I}}}_{P}={{\bf{I}}}_{P}^{(0)}+{\tilde{{\bf{A}}}}_{Q}\tilde{{\bf{Y}}}\tilde{{\bf{C}}}{\tilde{{\bf{Y}}}}^{-1}{[{\tilde{{\bf{I}}}}_{d}-{\tilde{{\bf{A}}}}_{T}\tilde{{\bf{Y}}}\tilde{{\bf{C}}}{\tilde{{\bf{Y}}}}^{-1}]}^{-1}{{\bf{I}}}_{Z}^{(0)}$$

In Eq. (25), ${{\bf{I}}}_{P}^{(0)}$ and ${{\bf{I}}}_{Z}^{(0)}$ represent the circuit response to the exciting voltage source without modulation. These matrices are easy to compute by LTI theory. The non-zero elements in ${{\bf{I}}}_{P}^{(0)}$ and ${{\bf{I}}}_{Z}^{(0)}$ are:

26 $$\begin{array}{c}{I}_{P1,0}^{(0)}={P}_{I}{U}_{1},{I}_{P2,0}^{(0)}={I}_{P3,0}^{(0)}={P}_{IJ}{U}_{1},\\ {I}_{Z11,0}^{(0)}={I}_{Z12,0}^{(0)}={R}_{I}{U}_{1},{I}_{Z21,0}^{(0)}={I}_{Z22,0}^{(0)}={I}_{Z31,0}^{(0)}={I}_{Z32,0}^{(0)}={R}_{IJ}{U}_{1}.\end{array}$$

The S-parameters for the proposed circulator can be obtained by

27 $${S}_{11}=1-2\frac{{I}_{P1,0}}{{U}_{1}}{Z}_{0},{S}_{21}=-\,2\frac{{I}_{P2,0}}{{U}_{1}}{Z}_{0},{S}_{31}=-\,2\frac{{I}_{P3,0}}{{U}_{1}}{Z}_{0}.$$

where *I*_*Pi*,0_ (*i* = 1, 2, 3) is the carrier frequency steady-current current at *i*-th port as defined in Table 2.

Equations (25)–(27) constitute the complete semi-analytical model of the proposed circulator as shown in Fig. 1. As evident from the derivation process, the model is agnostic of the LTI network and the switch performance and only needs linear matrices to describe the behaviour of these components. This is the most powerful aspect of this model, which dramatically simplifies the description of LPTV networks.

## Experimental Verification of the Circulator Model

**Figure 4:**
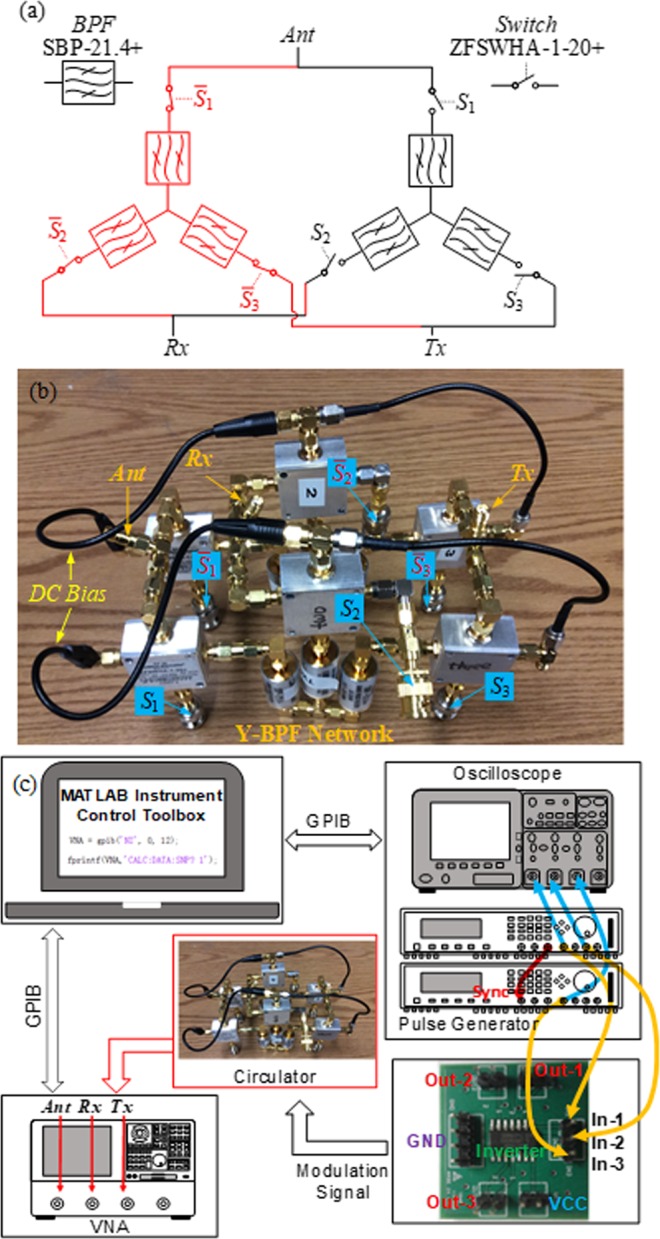
Schematic diagram and photo of the circulator and the experimental setup for the characterization of the circulator. (**a,b**) The schematic representation and the photo of the circulator architecture implemented by using MiniCitcuits discrete RF high isolation switches, ZFSWHA-1–20+, and bandpass filters (BPFs), SBP-21.4+ to validate the theoretical model. The modulation signals are the same as represented in Fig. 1. In this implementation, the LTI networks are Y-connected BPF networks. (**c**) Experimental setup for the characterization of the proposed circulator. For the inverter PCB, each Out-*k* (*k* = 1, 2, 3) is a pair of pins, carrying two signals, one of which is the same as In-*k* (*k* = 1, 2, 3) and the other is its complementary.

**Figure 5:**
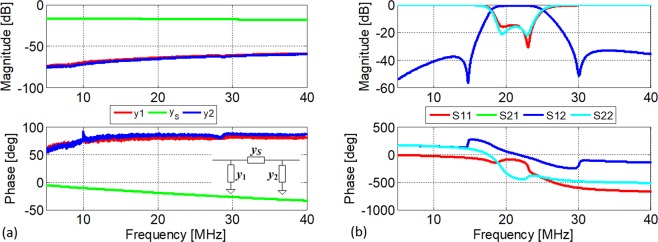
Response spectrum of the switch and filter used in the circulator circuit. (**a**) Measured frequency response of each of the element of the switch’s π-network equivalent model shown in the inset of the phase diagram. (**b**) Measured S-parameters of the selected filter, SBP-21.4+.

**Figure 6:**
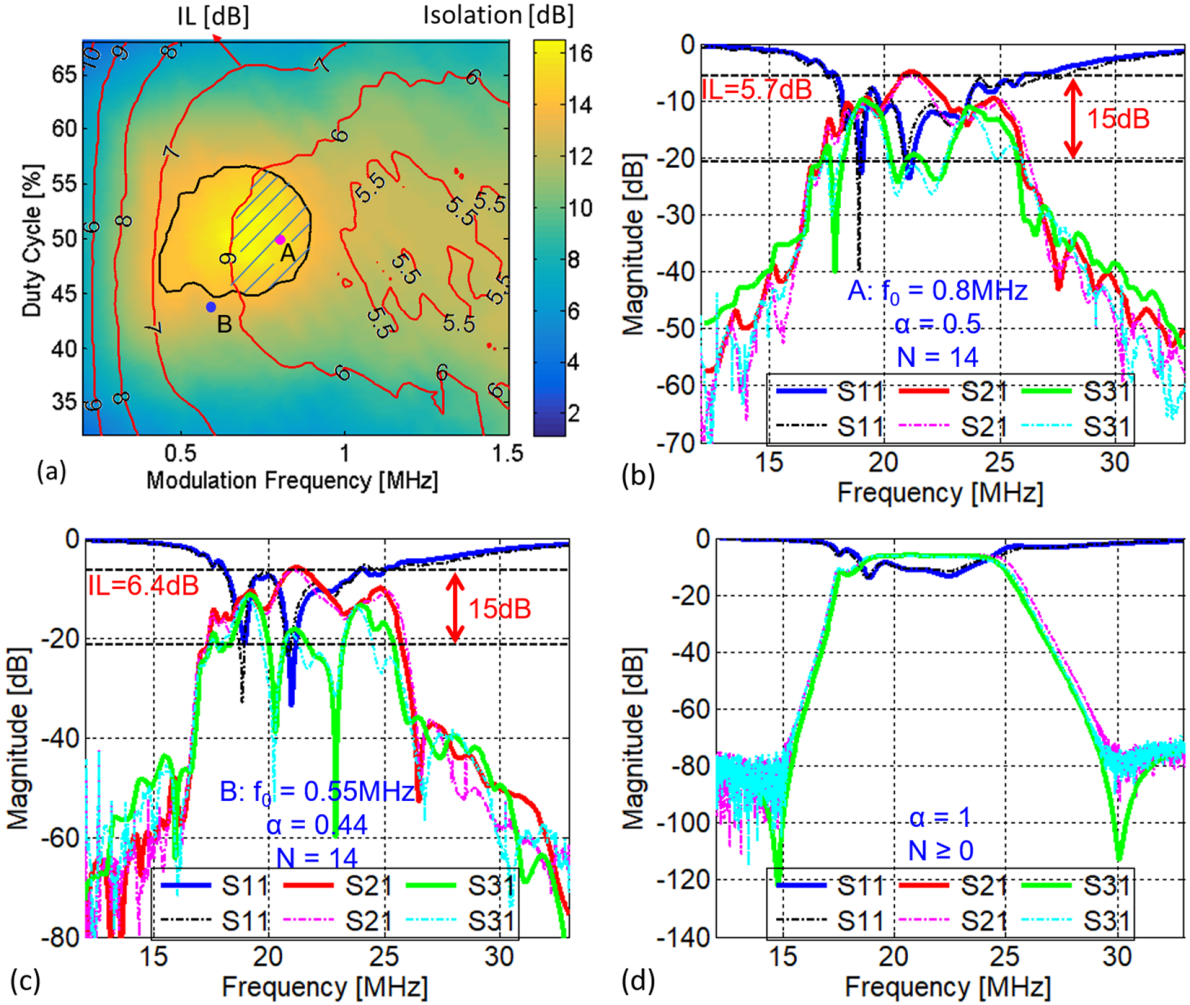
Circulator performance measurement results. (**a**) The performance in isolation (represented by colour) and IL (red contour lines) of the implemented circulator versus modulation frequency and duty cycle. The area within the black contour line is the parameter space that produces isolation larger than 14 dB, while the shaded area is the region that can offer *IL* lower than 6 dB at the same time. (**b**) The overlap between theoretical (solid lines) and experimental (dotted lines) responses of the circulator with modulation parameters (0.8 MHz, 50%) given by point A in (**a**). (**c**) The overlap between theoretical (solid lines) and experimental (dotted lines) responses of the circulator with modulation parameters (0.55 MHz, 44%) given by point B in (**a**). (**d**) The overlap between theoretical (solid lines) and experimental (dotted lines) responses of the circulator with duty cycle of 100%, in which one of the LTI network is off. The IL at 21.4 MHz is 6.52 dB. These losses are due to power splitting (3.52 dB), the IL of two BPF filters in series (1.62 dB) and two series switches (2.84 dB). The remaining 0.16 dB comes from the connectors.

To verify the previously described circulator model, we set up a circulator circuit as shown in Fig. 4a and b by using MiniCitcuits discrete RF high isolation switches, ZFSWHA-1-20+, and bandpass filters (BPF), SBP-21.4+. Figure 5a,b show the frequency responses of the selected switch and filter, respectively. The filter centre frequency is 21.4 MHz and its 3dB-bandwidth (BW) is 7.4 MHz. The insertion loss (IL) of the filter and switch at 21.4 MHz are 0.81 dB and 0.61 dB, respectively. As shown in Fig. 5a, since the magnitudes of *y*_1_ and *y*_2_ are at least 100 times smaller than *y*_*S*_, it is reasonable to neglect their shunt components in the model of the switch and only take its series admittance, *y*_*S*_, into account. As for the modulation signals, three pulse trains were generated by two synchronized 2-output pulse generators, Agilent 81110 A, which are then fed to a hex inverter, 74HCT04, to generate 3 complementary pairs of square waves, as shown in Fig. 4c. The phase differences between these signals were monitored by a 4-channel oscilloscope, Agilent DSO6014, and are automatically adjusted to be 120° by a MATLAB program. By sweeping the modulation frequency and duty cycle of the signal applied to the switches, we obtained a family of responses for the circulator. Figure 6a shows an overlapped contour map between circulator isolation and IL for different modulation characteristics. The shaded area is the region where modulation parameters are chosen to offer both good isolation and IL. It can be concluded that trade-offs between IL and isolation exist because the best IL and the largest isolation occur for different modulation parameters. We are not interested in explaining the reasons behind these trade-offs, which can be found in C. Xu *et al*.^31^, but rather focus on the validation of the proposed theoretical model. We chose two example points, *i*.*e*. Point A and B in Fig. 6a, to compare the theoretical and experimental results, as shown in Fig. 6b and c, respectively. Point A is an example of the circulator operating in the shaded region, which offers IL of 5.7 dB and isolation of ~15 dB over a 3-dB bandwidth of ~1.5 MHz. Point B is another set of parameters providing slightly lower IL (6.4 dB) yet much larger isolation (>30 dB) for some particular frequencies. For both cases, *S*_11_ is well below −10 dB. As a corner case, Fig. 6d shows the circulator response for *α* = 1, which effectively turns on only the normal set of switches (black ones in Fig. 1a) and thus blocks the complementary branch of LTI network. This configuration is reciprocal since the switches are kept either on or off and thus no modulation effect is imparted to the networks. The reciprocity is validated by *S*_21_ = *S*_31_.

It is observed that the theoretical results overlap well with the experimental ones in all three cases (Fig. 6b–d). To guarantee such good agreement between theory and experiment for the first two cases (Fig. 6b,c), a maximum harmonic index *N* = 14 is used in this work, which is slightly greater than BW/*ω*_0_ (equal to 9.3 and 13.7 for Point A and B, respectively). Minor discrepancies exist between the model and experiments. These originates from the deviation of the circulator from 3-fold rotational symmetry due to differences in the BPFs transfer function as evident from the measured *S*_21_ and *S*_31_ shown in Fig. 6d.

**Figure 7:**
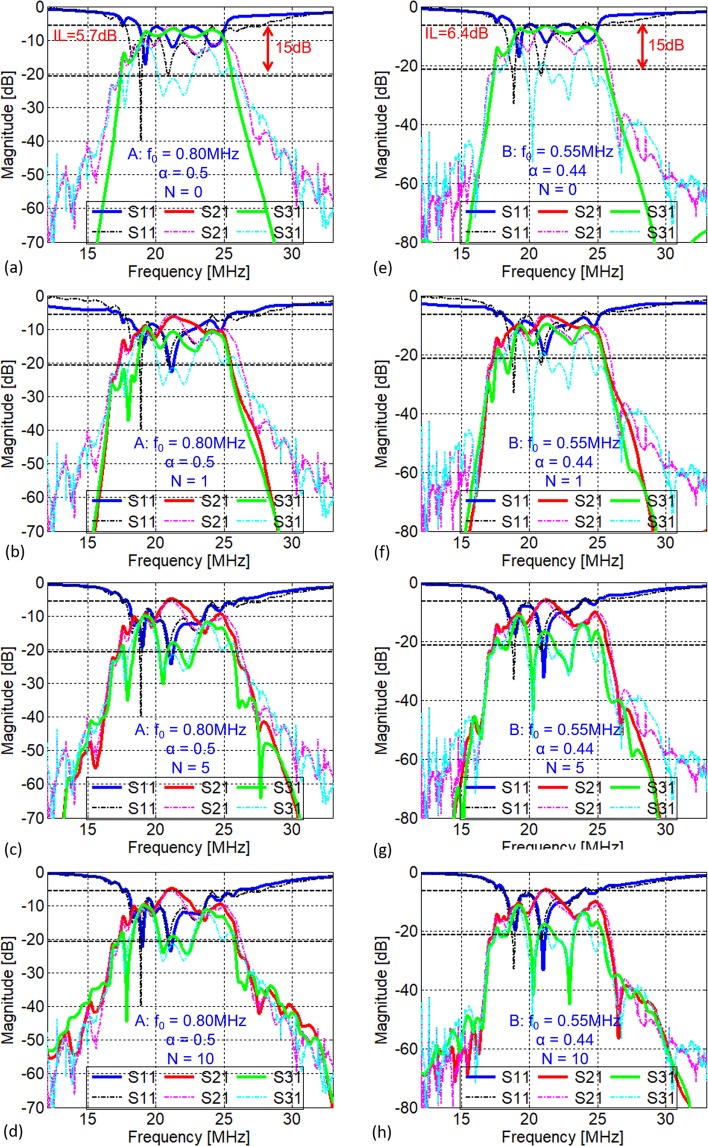
Comparison between theoretical (solid lines) and experimental (dotted lines) responses of the circulator for different *N* values and two sets of modulation parameters. *N* = 0, 1, 5, and 10. The modulation parameters for (**a**–**d**) and (**e**–**h**) are given by Point A Point B in Fig. 6a, respectively.

Figure 7 shows how the choice of smaller values of *N* (=0, 1, 5, 10) affects the prediction accuracy of the proposed circulator model. In particular, *N* = 0 (Fig. 7a,e) means no intermodulation products are accounted for, which essentially represents no modulation of the circulator. Therefore, Fig. 7a,e have the same theoretical response, in which *S*_21_ = *S*_31_, regardless of the modulation parameters. As expected, *N* = 1, *i*.*e*. only *ω*_*RF*_ and *ω*_*RF*_ ± *ω*_0_ being taken into consideration, cannot accurately predict the circulator behaviours, as shown in Fig. 7b,f. It is also interesting to note that, *N* = 5 is large enough to predict the in-band performance of the circulator, because the magnitudes of the intermodulation products are proportional to 1/*n*. By comparing Fig. 7c,d and Fig. 7g–h (*N* = 5, 10) to Fig. 6b,c (*N* = 14), respectively, it is worth noting that increasing *N* improves the accuracy in predicting the out-of-band response of the circulator. Interestingly, for circulator operated at Point A, the prediction with *N* = 10 (Fig. 7c) has no significant difference from that with *N* = 14 (Fig. 6b). Hence, selecting *N* slightly greater than BW/*ω*_0_ should be considered as a general rule of thumb to obtain an accurate description of the circulator network. As for the third case where *α* = 1 (Fig. 6d), any *N* ≥ 0 produces the same results from the circulator model as expected from Eqs (4) and (5) Further details of reproducing plots herein can be referred to Supplementary Information for “A Generalized Model for Linear-Periodically-Time-Variant Circulators”.

It is worth noting that the model does not make any assumption on the nature of the control signals. For example, the clock signal of any switch overlaps with that of two others at any given time in both cases that were analysed. Furthermore, even two different duty cycles (44% and 56%) were used for the case in Point B. This further proves the power of our generalized circulator model.

In conclusion, we have proven the validity of our circulator model, which can be used to predict circulator behaviours accurately, given specific switch performance, LTI response, modulation frequency, and duty cycle. It should be emphasized that no particular LTI network property is used in the derivation of the circulator model. Therefore, the model is broadly valid for all LTI networks with 3-fold symmetry. Moreover, it is easy to extend the model to account for general LTI networks and arbitrary switch configurations. It is also worth pointing out that a simplified model of the switch was used in this theoretical analysis for the experimental validation of the circulator model. A perfect open was used to model the switch in the off state and shunt parasitics were neglected in both the on and off states. Although beyond the scope of this work, the finite off-impedance and shunt parasitic can be separated from the switch and treated as part of LTI networks so that no modification to the form of the semi-analytical model is needed. Similarly, the switching speed was assumed to be infinite, but this is not a requirement for the proposed methodology. Any real switch signals in the time-domain can be described by complex Fourier transform coefficients (as explained in Methods section), hence easily incorporating the impact of non-ideal switching into the model.

## Conclusions

This work rigorously derived and experimentally validated a generalized frequency-domain semi-analytical model for LPTV circulators. The overall model is enabled by describing the behaviour of the switch using a resistor in parallel with a CCCS. With the help of the switch model, the analysis of LPTV circuits can be reduced to the linear superposition of LTI circuits. Although applied to a particular circulator network, the proposed model can be used to describe any LPTV circulators.

## Methods

**Figure 8:**
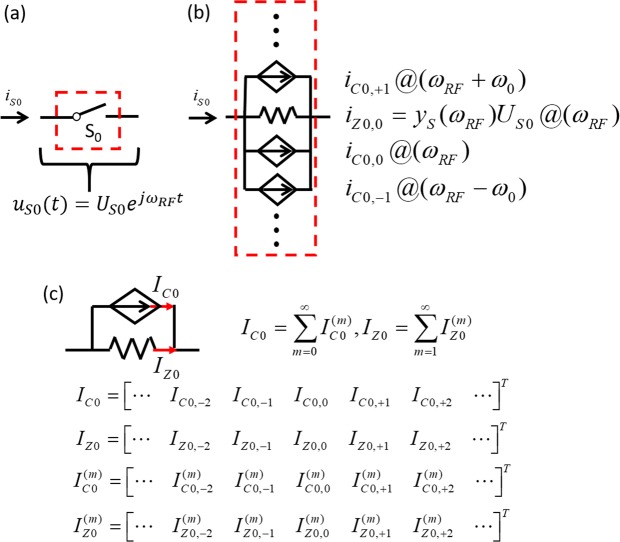
Schematic representation of the flow process used to the derive the equivalent circuit of a switch. (**a**) Current is induced by a voltage across the switch at the initial iteration. (**b**) The equivalent circuit of the switch formed by a resistor of *z*_*S*_(*ω*) = 1/*y*_*S*_(*ω*) Ohms and infinite CCCSs at the initial iteration. (**c**) The equivalent circuit of the switch formed by a resistor of *z*_*S*_(*ω*) = 1/*y*_*S*_(*ω*) Ohms and a CCCS accounting for infinite number of harmonics and iterations.

**Figure 9:**
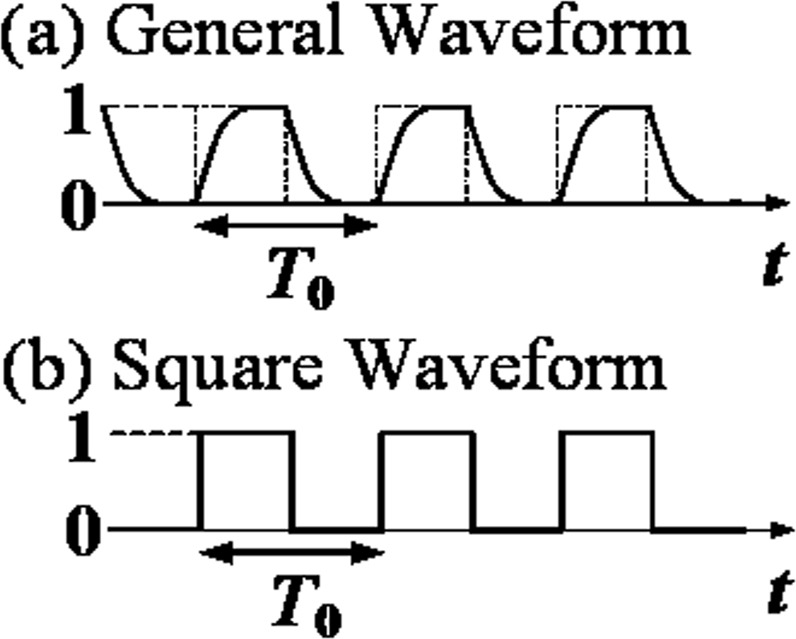
Schematic representation of the switching behaviour of the switch in time domain toggling between normalized amplitudes of 1 (ON) and 0 (OFF). (**a**) general waveform and (**b**) square waveform.

The switch behaviour is described by building a model of its I-V relationship under modulation. In the off-state, the switch presents a small admittance when in the off-state and a large one, *y*_*S*_(*ω*), when in the on-state. Since a switch with non-zero off-admittance can be treated as equivalent to a zero off-admittance switch in parallel with a component that has the same admittance of the switch’s off-admittance incorporated in the LTI network, we can consider the switch off-state admittance to be zero without imposing any particular restrictions on the validity of the model. Therefore, without loss of generality, we can assume that the switch admittance spectrum is equal to *y*_*S*_(*ω*) Siemens when in on-state and 0 Siemens when in off-state. When a voltage of *u*_*S*0_(*t*) = *U*_*S*0_exp(*jω*_*RF*_*t*) at the carrier frequency, *ω*_*RF*_, is applied across the switch (Fig. 8a), the corresponding excited steady-state current, *i*_*S*0_ (assuming *i*_*S*0_ exists), through the switch in the time domain is

28 $${i}_{S0}(t)={\Im }^{-1}[{y}_{S}(\omega )]\otimes y(t){u}_{S0}(t)$$

where ℑ^−1^(⋅) represents the inverse Fourier transform, ⊗ represents the convolution operator, and *y*(*t*) is the switch’s time-domain behaviour toggling between normalized ON, *y*(*t*) = 1, and OFF, *y*(*t*) = 0, as shown in Fig. 9a. *y*(*t*) can be represented by a complex Fourier series as

29 $$y(t)=\sum _{n=-\infty }^{+\infty }\,{c}_{n}{e}^{-jn\theta }\,{e}^{jn{\omega }_{0}t}$$

where *θ* is the phase delay of the modulation signal, *c*_*n*_ is the *n*-th coefficient of the complex Fourier transform of the general periodic switching behaviour in time-domain. Without loss of generality in deriving the switch model, an ideally sharp switching behaviour, *i*.*e*. a square wave (Fig. 9b), is used to describe *y*(*t*) in this work so that:

30 $$y(t)=rect(\frac{t}{\alpha {T}_{0}}-\frac{1}{2})\otimes \sum _{n=-\infty }^{+\infty }\,\delta (t-n{T}_{0}-\tau ),$$

where *α* is the duty cycle, and *τ* = *T*_0_∙*θ*/(2*π*) is the time delay of the modulation signal. In this case, *c*_*n*_ = (1 − *e*^−*j*2*αnπ*^)/(*j*2*nπ*) and particularly, *c*_0_ = *α*.

Combining Eqs (28) and (29) produces,

31 $${i}_{S0}(t)={i}_{Z0,0}{e}^{j{\omega }_{RF}t}+\sum _{n=-\infty }^{+\infty }\,{i}_{C0,n}{e}^{j({\omega }_{RF}+n{\omega }_{0})t}$$

where *i*_*Z*0,0_ = *y*_*S*_(*ω*_*RF*_)*U*_*S*0_, *i*_*C*0,*n*_ = *y*_*S*_(*ω*_*RF*_ + *nω*_0_)(*e*^−*jnθ*^*c*_*n*_)*z*_*S*_(*ω*_*RF*_)*i*_*Z*0,0_(*n* ≠ 0), *i*_*C*0,0_ = (*c*_0_ − 1)*i*_*Z*0,0_. From Eq. (31), infinite intermodulation products (*ω*_*RF*_ ± *nω*_0_) are generated due to the mixing effect. The first term ${i}_{Z0,0}\exp (j{\omega }_{RF}t)$ obeys Ohm’s law as if a voltage *U*_*S*0_exp(*jω*_*RF*_*t*) was applied across an equivalent resistor of *z*_*S*_(*ω*) Ohms, while the rest can be treated as current controlled current sources (CCCSs) with a signal equal to ${i}_{C0,n}\exp [j({\omega }_{RF}+n{\omega }_{0})t]$ dependent on *i*_*Z*0,0_ at the intermodulation frequencies, (*ω*_*RF*_ + *nω*_0_), in parallel with the equivalent resistance of the switch, as shown in Fig. 8b. Equation (31) also implies that once there is current ${i}_{Z0,n}\exp [j({\omega }_{RF}+n{\omega }_{0})t]$ flowing through the equivalent resistor, CCCSs are generated accordingly. Upon the interaction of current sources with external circuits, the current sources inject currents into the equivalent resistor, then causing another iteration of mixing process. This process repeats until the circuit currents and voltages converge at steady state. In this state, there are infinite intermodulation frequency currents flowing through the equivalent resistor in parallel with infinite intermodulation frequency CCCSs, which are represented in a compact form as shown in Fig. 8c. For convenience, the vectors comprised of current phasors (frequency information is omitted but implied by the positions and subscripts of phasors) are used to represent the circuit states. In this work, we call them phasor vectors. The steady-state currents, **I**_Z0_ and **I**_C0_, can be decomposed into the sum of infinite iterative currents, ${{\bf{I}}}_{Z0}^{(m)}$, and ${{\bf{I}}}_{C0}^{(m)}$, respectively, where *m* represents the iteration number.

**Figure 10:**
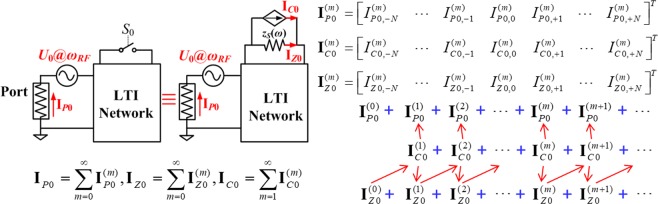
Schematic diagram showing the switch in an arbitrary LTI circuit, its corresponding equivalent circuit and the interaction between currents in the circuit. I_*P*0_, I_*Z*0_, and I_*C*0_ are the steady-state currents of the port, the equivalent resistor, and the CCCS, respectively. Each current is the sum of its corresponding iterative currents, ${{\bf{I}}}_{P0}^{(m)}$, ${{\bf{I}}}_{Z0}^{(m)}$, or ${{\bf{I}}}_{C0}^{(m)}$. ${{\bf{I}}}_{P0}^{(0)}$ and ${{\bf{I}}}_{Z0}^{(0)}$ are the circuit response without modulation. The red arrows on the right represent the iteration flow of the mixing process. It is important to note that all components of ${{\bf{I}}}_{P0}^{(0)}$ and ${{\bf{I}}}_{Z0}^{(0)}$ are zero except their elements at carrier frequency, which are represented by ${I}_{P0,0}^{(0)}$ and ${I}_{Z0,0}^{(0)}$, respectively.

Now, let us consider the interaction between a switch and a general LTI electrical network, which is driven by a voltage source, as shown in Fig. 10. The circuit states can be completely described by phasor vectors, **I**_*P*0_, **I**_*Z*0_, and **I**_*C*0_, which are the sum of all the corresponding iterative currents, ${{\bf{I}}}_{P0}^{(m)}$, ${{\bf{I}}}_{Z0}^{(m)}$, and ${{\bf{I}}}_{C0}^{(m)}$, respectively. As explained in the main manuscript, it is not necessary to compute all infinite number of intermodulation terms in the circuit state vectors. Therefore, in Fig. 10, only (2*N* + 1) intermodulation frequencies, *i.e*. from (*ω*_*RF*_ − *Nω*_0_) to (*ω*_*RF*_ + *Nω*_0_), are taken into account. Fig. 10 also shows the correlation between iterative currents generated by the frequency mixing due to the switch modulation. To express the modulation effect, rewriting Eq. (31) in a matrix form produces

32 $${{\bf{I}}}_{C0}^{(m+1)}={\bf{YC}}(\alpha ,\theta ){{\bf{Y}}}^{-1}{{\bf{I}}}_{Z0}^{(m)}$$

where **Y** is a diagonal admittance matrix related to the switch’s on-admittance, *y*_*S*_(*ω*) = 1/*z*_*S*_(*ω*), which is given by

33 $$\begin{array}{rcl}{\bf{Y}} & = & {\bf{Y}}({\omega }_{RF},{\omega }_{0})\\  & = & diag[{y}_{S0}({\omega }_{RF}-N{\omega }_{0}),\ldots ,\,{y}_{S0}({\omega }_{RF}-{\omega }_{0}),\,{y}_{S0}({\omega }_{RF}),\,{y}_{S0}({\omega }_{RF}+{\omega }_{0}),\ldots ,\,{y}_{S0}({\omega }_{RF}+N{\omega }_{0})]\end{array}$$

and **C**(*α*, *θ*) is a (2*N* + 1)-order matrix dictating the mapping relationship in frequency conversion, which is expressed as

34 $${\bf{C}}(\alpha ,\theta )=[\begin{array}{cccccc}{c}_{0}-1 & {c}_{-1}{e}^{j\theta } & \cdots  & {c}_{-k}{e}^{jk\theta } & \cdots  & {c}_{-2N}{e}^{j(2N)\theta }\\ {c}_{1}{e}^{-j\theta } & {c}_{0}-1 & \cdots  & {c}_{-k+1}{e}^{j(k-1)\theta } & \cdots  & {c}_{-2N+1}{e}^{j(2N-1)\theta }\\ \vdots  & \vdots  & \ddots  & \vdots  & \cdots  & \vdots \\ {c}_{l}{e}^{-l\theta } & {c}_{l-1}{e}^{-j(l-1)\theta } & \cdots  & {C}_{lk}(\alpha ,\theta ) & \cdots  & {c}_{-2N+l}{e}^{j(2N-l)\theta }\\ \vdots  & \vdots  & \cdots  & \vdots  & \ddots  & \vdots \\ {c}_{2N}{e}^{-j(2N)\theta } & {c}_{2N-1}{e}^{-j(2N-1)\theta } & \cdots  & {c}_{2N-k}{e}^{-j(2N-k)\theta } & \cdots  & {c}_{0}-1\end{array}],$$

where $${C}_{lk}(\alpha ,\theta )=\{\begin{array}{ll}{c}_{0}-1, & l=k\\ {c}_{l-k}{e}^{-j(l-k)\theta }, & l\ne k\end{array}$$. The second aspect depicted in Fig. 10 is the correlation between iterative currents as described by the transfer functions of the LTI network, *T*_0_(*ω*) and *Q*_0_(*ω*), defined in Fig. 2c,d. Expressing the current contribution from CCCS at either the equivalent resistor or the port in Fig. 2b leads to

35 $${{\bf{I}}}_{Z0}^{(m+1)}={{\bf{A}}}_{{T}_{0}}{{\bf{I}}}_{C0}^{(m+1)}$$

36 $${{\bf{I}}}_{P0}^{(m+1)}={{\bf{A}}}_{{Q}_{0}}{{\bf{I}}}_{C0}^{(m+1)}$$

where **A**_*T*0_ and **A**_*Q*0_ represent absorption matrices that depict the ability of the switch equivalent resistor and termination port to absorb current from CCCS at different intermodulation frequencies, respectively. They are written as

37 $$\begin{array}{rcl}{{\bf{A}}}_{{T}_{0}} & = & {{\bf{A}}}_{{T}_{0}}({\omega }_{RF},{\omega }_{0})\\  & = & diag\,[{T}_{0}({\omega }_{RF}-N{\omega }_{0}),\ldots ,\,{T}_{0}({\omega }_{RF}-{\omega }_{0}),{T}_{0}({\omega }_{RF}),\,{T}_{0}({\omega }_{RF}+{\omega }_{0}),\ldots ,\,{T}_{0}({\omega }_{RF}+N{\omega }_{0})].\end{array}$$

38 $$\begin{array}{rcl}{{\bf{A}}}_{{Q}_{0}} & = & {{\bf{A}}}_{{Q}_{0}}({\omega }_{RF},{\omega }_{0})\\  & = & diag[{Q}_{0}({\omega }_{RF}-N{\omega }_{0}),\ldots ,\,{Q}_{0}({\omega }_{RF}-{\omega }_{0}),\,{Q}_{0}({\omega }_{RF}),\,{Q}_{0}({\omega }_{RF}+{\omega }_{0}),\ldots ,\,{Q}_{0}({\omega }_{RF}+N{\omega }_{0})].\end{array}$$

Taking the summation of Eq. (32) from *m* = 0 to *m* = *M* gives

39 $$\sum _{m=0}^{M}\,{{\bf{I}}}_{C0}^{(m+1)}=\sum _{m=0}^{M}\,{\bf{YC}}(\alpha ,\theta ){{\bf{Y}}}^{-1}{{\bf{I}}}_{Z0}^{(m)}$$

By taking the limit when *M* approaches infinity as well as combining ${{\rm{I}}}_{Z0}=\sum _{m=0}^{\infty }\,{{\rm{I}}}_{Z0}^{(m)}$ and ${{\rm{I}}}_{C0}=\sum _{m=1}^{\infty }\,{{\rm{I}}}_{C0}^{(m)}$, Eq. (2) is obtained. In particular, if the switch has a constant spectral on-impedance (including ideal zero-resistance), Eq. (2) can be simplified as

40 $${{\bf{I}}}_{C0}={\bf{C}}(\alpha ,\theta ){{\bf{I}}}_{Z0}$$

Similarly, taking the summation of Eq. (35) from *m* = 0 to *m* = *M* produces

41 $$\sum _{m=0}^{M}\,{{\bf{I}}}_{Z0}^{(m+1)}=\sum _{m=0}^{M}\,{{\bf{A}}}_{{T}_{0}}{{\bf{I}}}_{C0}^{(m+1)}$$

Adding ${{\bf{I}}}_{Z0}^{(0)}$ to both sides of Eq. (41) and taking the limit when *M* approaches infinity, we obtain Eq. (6). Again, taking the summation of Eq. (36) fro*m m* = 0 to *m* = *M* produces

42 $$\sum _{m=0}^{M}\,{{\bf{I}}}_{P0}^{(m+1)}=\sum _{m=0}^{M}\,{{\bf{A}}}_{{Q}_{0}}{{\bf{I}}}_{C0}^{(m+1)}$$

Adding ${{\bf{I}}}_{P0}^{(0)}$ to both sides of Eq. (42), taking the limit when *M* approaches infinity, and applying ${{\rm{I}}}_{P0}=\sum _{m=0}^{\infty }\,{{\rm{I}}}_{P0}^{(m)}$, we have Eq. (7).

## Supplementary information

Supplementary Information for “A Generalized Model for Linear-Periodically-Time-Variant Circulators”

Dataset 1

Dataset 2

Dataset 3A

Dataset 3B

## Data Availability

Data presented in this work will be made available by the authors upon appropriate request.
