# Supplementary material for: A Generalized Model for Linear-Periodically-Time-Variant Circulators
Source: Sci Rep. 2019 Jun 18;9:8718. doi: 10.1038/s41598-019-45013-5 (PMC6582053; doi:10.1038/s41598-019-45013-5)
Supplement: Supplementary file 1 — Supplementary Information for “A Generalized Model for Linear-Periodically-Time-Variant Circulators” [file 41598_2019_45013_MOESM1_ESM.docx]

Supplementary Information for

“A Generalized Model for Linear-Periodically-Time-Variant Circulators”

Changting Xu^[[1]](#footnote-1)^‡, Gianluca Piazza^[[2]](#footnote-2)^*

This supplementary material contains the MATLAB codes that are used to predict the S-parameters of the proposed circulator in Fig. 4. Before running the circulator_calc function, *p*- and *t*-functions and on-admittance of RF switches need to be calculated or simulated by circuit analysis tools over a large enough frequency range, [*f_L_*, *f_H_*]. These data are saved in two files: Net.xlsx and Switch.xlsx. By ignoring the shunt paths of switches, *i.e.* *y*_1_ and *y*_2_ in Fig.5, *r*- and *q*-functions can be derived from *p*- and *t*-functions via Kirchhoff's Current Law (KCL), respectively. Hence, *r*- and *q*-functions are neither simulated in advance nor explicitly declared in the codes. Assuming the sweep frequency of interest is [*f*_1_, *f*_2_], the maximum modulation frequency, *f*_0_, and the maximum mixing order, *N*, then the simulation frequency range must satisfy *f_L_* ≤ *f*_1_ – *N∙f*_0_, *f_H_* ≥ *f*_2_ + *N∙f*_0_ to avoid singular matrix issues. Data are loaded by the MATLAB code, followed by the calculation of matrix $\tilde{\mathbf{C}}, \tilde{\mathbf{Y}}, {\tilde{\mathbf{A}}}_{T}$ and ${\tilde{\mathbf{A}}}_{Q}$. The corresponding circuit steady state currents, $\mathbf{I}_{Z}$ and $\mathbf{I}_{P}$, are computed by equations (24), (17), and (19). The same process is repeated for every frequency of interest. Finally, S-parameters can be obtained via equation (27). Due to the assumption of 3-fold rotational symmetry, only *S*_11_, *S*_21_, and *S*_31_ need to be calculated.

**MATLAB Code**

function [S11,S21,S31] = circulator_calc(Net,Switch,f_sweep,fm,M_order,alpha,Z0)

% Net: table containing PI,PIJ,TI1,TIJ1,TI2,TIJ2

% Switch: table containing y1,y2,ys

% f_sweep: sweep frequency of interest in MHz, column vector

% fm: modulation frequency in MHz

% M_order: mixing order of modulation frequency

% alpha: duty cycle

% Z0: characteristic impedance

theta = (0:2)*2/3*pi; % phase delays in modulation signals

f = @(n,th) exp(-1j*n*th).*(1-exp(-1j*2*alpha*n*pi))./(1j*2*n*pi);

f_bar = @(n,th) exp(-1j*n*(2*alpha*pi+th)).*(1-exp(-1j*2*(1-alpha)*n*pi))./(1j*2*n*pi);

freq_num = 2*M_order + 1; % the number of mixing frequency of interest

index_shift = M_order + 1; % index of the carrier freq

% construction of C_MATRIX

C1_matrix = zeros(freq_num);

C2_matrix = zeros(freq_num);

C3_matrix = zeros(freq_num);

C1_matrix_bar = zeros(freq_num);

C2_matrix_bar = zeros(freq_num);

C3_matrix_bar = zeros(freq_num);

for p = 1:size(C1_matrix,2)

C1_matrix(:,p) = f((0:2*M_order)'-(p-1), theta(1));

C2_matrix(:,p) = f((0:2*M_order)'-(p-1), theta(2));

C3_matrix(:,p) = f((0:2*M_order)'-(p-1), theta(3));

C1_matrix_bar(:,p) = f_bar((0:2*M_order)'-(p-1), theta(1));

C2_matrix_bar(:,p) = f_bar((0:2*M_order)'-(p-1), theta(2));

C3_matrix_bar(:,p) = f_bar((0:2*M_order)'-(p-1), theta(3));

end

C1_matrix(eye(freq_num)~=0) = -(1-alpha); % replace the diagonal NAN with -(1-alpha)

C2_matrix(eye(freq_num)~=0) = -(1-alpha); % note the minus sign

C3_matrix(eye(freq_num)~=0) = -(1-alpha);

C1_matrix_bar(eye(freq_num)~=0) = -alpha; % replace the diagonal NAN with -alpha

C2_matrix_bar(eye(freq_num)~=0) = -alpha; % note the minus sign

C3_matrix_bar(eye(freq_num)~=0) = -alpha;

C_MATRIX = zeros(6*freq_num);

C_MATRIX(1:freq_num,1:freq_num) = C1_matrix;

C_MATRIX((1:freq_num)+freq_num,(1:freq_num)+freq_num) = C1_matrix_bar;

C_MATRIX((1:freq_num)+2*freq_num,(1:freq_num)+2*freq_num) = C2_matrix;

C_MATRIX((1:freq_num)+3*freq_num,(1:freq_num)+3*freq_num) = C2_matrix_bar;

C_MATRIX((1:freq_num)+4*freq_num,(1:freq_num)+4*freq_num) = C3_matrix;

C_MATRIX((1:freq_num)+5*freq_num,(1:freq_num)+5*freq_num) = C3_matrix_bar;

% interpolation of p- and t-functions, series admittance ys

PI_matrix = zeros(freq_num, length(f_sweep));

PIJ_matrix = zeros(freq_num, length(f_sweep));

TI1_matrix = zeros(freq_num, length(f_sweep));

TIJ1_matrix = zeros(freq_num, length(f_sweep));

TI2_matrix = zeros(freq_num, length(f_sweep));

TIJ2_matrix = zeros(freq_num, length(f_sweep));

ys_matrix = zeros(freq_num, length(f_sweep));

for i = 1:freq_num

PI_matrix(i,:) = interp1(Net.f_MHz, Net.PI, f_sweep + (i-index_shift)*fm);

PIJ_matrix(i,:) = interp1(Net.f_MHz, Net.PIJ, f_sweep + (i-index_shift)*fm);

TI1_matrix(i,:) = interp1(Net.f_MHz, Net.TI1, f_sweep + (i-index_shift)*fm);

TIJ1_matrix(i,:) = interp1(Net.f_MHz, Net.TIJ1, f_sweep + (i-index_shift)*fm);

TI2_matrix(i,:) = interp1(Net.f_MHz, Net.TI2, f_sweep + (i-index_shift)*fm);

TIJ2_matrix(i,:) = interp1(Net.f_MHz, Net.TIJ2, f_sweep + (i-index_shift)*fm);

ys_matrix(i,:) = interp1(Switch.f_MHz, Switch.ys, f_sweep + (i-index_shift)*fm);

end

% Port current declaration

curr_port1 = zeros(freq_num, 2, length(f_sweep));

curr_port2 = zeros(freq_num, 2, length(f_sweep));

curr_port3 = zeros(freq_num, 2, length(f_sweep));

% Switch current declaration

curr_switch11 = zeros(freq_num, 2, length(f_sweep));

curr_switch21 = zeros(freq_num, 2, length(f_sweep));

curr_switch31 = zeros(freq_num, 2, length(f_sweep));

curr_switch12 = zeros(freq_num, 2, length(f_sweep));

curr_switch22 = zeros(freq_num, 2, length(f_sweep));

curr_switch32 = zeros(freq_num, 2, length(f_sweep));

% the first column: initial current with modulation

% the second column: final current with modulation

% assignment of the first column by equation (26)

curr_port1(index_shift,1,:) = interp1(Net.f_MHz, Net.PI, f_sweep);

curr_port2(index_shift,1,:) = interp1(Net.f_MHz, Net.PIJ, f_sweep);

curr_port3(index_shift,1,:) = curr_port2(index_shift,1,:);

curr_switch11(index_shift,1,:) = curr_port1(index_shift,1,:)/2;

curr_switch21(index_shift,1,:) = curr_port2(index_shift,1,:)/2;

curr_switch31(index_shift,1,:) = curr_port2(index_shift,1,:)/2;

curr_switch12(index_shift,1,:) = curr_port1(index_shift,1,:)/2;

curr_switch22(index_shift,1,:) = curr_port2(index_shift,1,:)/2;

curr_switch32(index_shift,1,:) = curr_port2(index_shift,1,:)/2;

% calculation of the final current

for r = 1:length(f_sweep) % loop for different frequency

Y_tilde = zeros(6*freq_num);

Y = diag(ys_matrix(:,r));

for count = 1:6

Y_tilde((1:freq_num)+(count-1)*freq_num,(1:freq_num)+(count-1)*freq_num) = Y;

end

YCY_MATRIX = (Y_tilde*C_MATRIX)/Y_tilde;

A_TI1 = diag(TI1_matrix(:,r));

A_TIJ1 = diag(TIJ1_matrix(:,r));

A_TI2 = diag(TI2_matrix(:,r));

A_TIJ2 = diag(TIJ2_matrix(:,r));

A_TI = [A_TI1, A_TI2;

A_TI2, A_TI1];

A_TIJ = [A_TIJ1, A_TIJ2;

A_TIJ2, A_TIJ1];

A_tilde_T = [A_TI, A_TIJ, A_TIJ;

A_TIJ, A_TI, A_TIJ;

A_TIJ, A_TIJ, A_TI];

A_tilde_Q = zeros(3*freq_num, 6*freq_num);

Q_MATRIX_EYE = eye(6*freq_num) + A_tilde_T;

A_tilde_Q(1:freq_num,:) = Q_MATRIX_EYE(1:freq_num,:) + ...

Q_MATRIX_EYE((1:freq_num)+freq_num,:);

A_tilde_Q((1:freq_num)+freq_num,:) = Q_MATRIX_EYE((1:freq_num)+2*freq_num,:) + ...

Q_MATRIX_EYE((1:freq_num)+3*freq_num,:);

A_tilde_Q((1:freq_num)+2*freq_num,:) = Q_MATRIX_EYE((1:freq_num)+4*freq_num,:) + ...

Q_MATRIX_EYE((1:freq_num)+5*freq_num,:);

A_matrix_tmp = eye(size(A_tilde_T)) - A_tilde_T*YCY_MATRIX;

b_vector = [curr_switch11(:,1,r);

curr_switch12(:,1,r);

curr_switch21(:,1,r);

curr_switch22(:,1,r);

curr_switch31(:,1,r);

curr_switch32(:,1,r)];

p_vector = [curr_port1(:,1,r);

curr_port2(:,1,r);

curr_port3(:,1,r)];

Xd_vector = A_matrix_tmp\b_vector; % equation (24)

curr_switch11(:,2,r) = Xd_vector(1:freq_num);

curr_switch12(:,2,r) = Xd_vector(freq_num+1:2*freq_num);

curr_switch21(:,2,r) = Xd_vector(2*freq_num+1:3*freq_num);

curr_switch22(:,2,r) = Xd_vector(3*freq_num+1:4*freq_num);

curr_switch31(:,2,r) = Xd_vector(4*freq_num+1:5*freq_num);

curr_switch32(:,2,r) = Xd_vector(5*freq_num+1:6*freq_num);

Xp_vector = p_vector + A_tilde_Q*YCY_MATRIX*Xd_vector; % equation (17) and (19)

curr_port1(:,2,r) = Xp_vector(1:freq_num);

curr_port2(:,2,r) = Xp_vector(freq_num+1:2*freq_num);

curr_port3(:,2,r) = Xp_vector(2*freq_num+1:3*freq_num);

end

Ip1 = reshape(curr_port1(index_shift,2,:),1,[]);

Ip2 = reshape(curr_port2(index_shift,2,:),1,[]);

Ip3 = reshape(curr_port3(index_shift,2,:),1,[]);

% calculation of S-parameters

S11 = 1-2*Ip1*Z0;

S21 = -2*Ip2*Z0;

S31 = -2*Ip3*Z0;

end

1. ‡ *QCT Stargate R&D – Santa Clara, Qualcomm Technologies, Inc., Santa Clara, CA, USA, 95051. (email: changtix@alumni.cmu.edu)* [↑](#footnote-ref-1)
2. * *Department of Electrical and Computer Engineer, Carnegie Mellon University, Pittsburgh, PA, USA, 15213. (email: piazza@ece.cmu.edu)* [↑](#footnote-ref-2)
